# “Bridging tradition and modern care: a narrative review on managing menopause in Saudi primary healthcare settings”

**DOI:** 10.3389/frph.2026.1841414

**Published:** 2026-06-04

**Authors:** Malak A. Al Shammari

**Affiliations:** Department of Family and Community Medicine, College of Medicine, Imam, Abdulrahman Bin Faisal University, Dammam, Saudi Arabia

**Keywords:** cultural perceptions, menopause, primary healthcare, Saudi Arabia, telemedicine, traditional medicine, women's health

## Abstract

**Background:**

Menopause is a multifaceted biological, psychological, and social transition experienced by women worldwide. While global research describes variations in symptom prevalence and cultural interpretations, the Saudi context remains underexplored despite its unique cultural, religious, and social influences. Saudi women commonly report vasomotor, psychological, and musculoskeletal symptoms, yet intimate and urogenital concerns remain underreported due to cultural norms. Primary healthcare (PHC) services face documented gaps in training, readiness, and communication, further limiting comprehensive care for menopausal women.

**Objective:**

This narrative review examines the cultural, religious, psychological, and social dimensions of menopause among Saudi women, evaluates patient–provider interaction dynamics within PHC services, and highlights opportunities for integrating culturally appropriate management approaches.

**Methods:**

A narrative synthesis of national and international literature was conducted, drawing on studies related to menopausal symptoms, cultural perceptions, healthcare system readiness, traditional therapies, and patient–provider communication in Saudi Arabia.

**Results:**

Findings demonstrate that cultural and Islamic values strongly shape women's perceptions of menopause, coping strategies, and health-seeking behavior. Herbal and traditional remedies remain widely used, often preferred over hormone replacement therapy. Significant barriers affect PHC-level care, including limited provider training, gender dynamics influencing consultation comfort, lack of privacy, and the absence of national menopause management guidelines. Psychologically, many women experience mood changes, anxiety, and altered self-perception, while social factors—particularly family and marital dynamics—play a critical role in adjustment. Emerging community-based programs and telemedicine platforms offer promising culturally sensitive avenues to improve access and quality of care.

**Conclusion:**

Menopause among Saudi women is a complex biopsychosocial experience shaped by deep cultural and religious contexts. Current PHC services have notable gaps but also clear opportunities for improvement through clinical training, culturally competent communication, integration of safe traditional practices, and expansion of telemedicine. Developing national guidelines and community-based education initiatives is essential to enhance patient-centered, culturally congruent menopausal care in Saudi Arabia.

## 1 Background

Many definitions of menopause exist in the literature within endocrinological, psychological or social context of change. Typically, it is defined as the biological process that marks the end of a woman's reproductive years (1). It is officially defined as the point in time when a woman has gone 12 consecutive months without a menstrual period, not caused by other medical conditions (2).

Although it may occur at any stage in a biological female's life, it usually occurs between the ages of 45 and 55 (3). The most reported bothersome symptoms in women going through the physiological changes of menopause are hot flushes and sweating during nighttime (4). Other concerning symptoms in women going through menopause include a variety of differently experienced symptoms like bone pains, fatigue and difficultly in weight loss (3, 4). Mood swings and poor sleep and concentration have been repeatedly mentioned as well (4).

Symptoms experienced by menopausal females but are not commonly addressed are related to vaginal atrophy and decreased libido stressing their intimate relationships with their spouses further affecting mental health and psychological well-being (4, 5).

It is worth mentioning that perimenopausal ladies most commonly report these previously mentioned physical symptoms, while post-menopausal females do not commonly report these symptoms; instead, when prompted, they often express psychological concerns (4, 5). Despite this observation, a well-defined gap in the literature exists in focusing on general wellbeing with sole concentration on certain mental health conditions like depression often being the case (5, 6).

Globally, 51 years is the average age of menopause occurrence. The timing can vary by genetics, lifestyle, and socioeconomic factors (7). Due to the increased life expectancy of the human population and the increase in the aging population, the number of postmenopausal women is expected to rise reaching 1.2 billion by 2030 (8).

Saudi research indicates that the average age of menopause is slightly lower, ranging from 48 to 50 years, possibly due to cultural, nutritional, and environmental differences (9). The prevalence of menopausal symptoms such as hot flashes, mood changes, and sleep disturbances is high among Saudi women, similar to global trends, but symptom reporting may be influenced by social and cultural factors (9, 10).

Despite the obvious burden this condition has on females and health systems, little is actively done to address and prevent its sequalae (10). Simple clinical practices, such as proactively inquiring about symptoms and adopting a comprehensive approach, can significantly improve menopausal care.

The well-established differences in timing and influencing factors among Saudi women require further research to achieve a deeper understanding. This knowledge can support family physicians and primary healthcare providers in delivering more effective and culturally appropriate care. Exploring and understanding Saudi culture, popular beliefs, religious perspectives and social norms and how they impact the perceptions and health seeking behaviours of women in our society will no doubt significantly impact their health management and satisfaction. In this review, modern care refers to evidence-based, guideline-informed menopause management approaches delivered within a patient-centered primary healthcare framework. This includes the use of standardized clinical guidelines (e.g., National Institute for Health and Care Excellence (NICE), American College of Obstetricians and Gynecologists (ACOG), individualized risk–benefit assessment for hormone replacement therapy, incorporation of non-hormonal therapies, structured patient education, and shared decision-making. Modern care also encompasses system-level components such as provider training, continuity of care, integration of mental health support, and the use of digital health technologies such as telemedicine. Importantly, in this review, such integration is presented as a conceptual framework for culturally responsive care rather than an evidence-based conclusion.

The objective of this narrative review is to provide a comprehensive examination of menopause among Saudi women by integrating cultural, religious, psychological, and social perspectives with existing healthcare system practices.

## 2 Methods

This narrative review was conducted using a structured approach to enhance transparency and minimize selection bias. A comprehensive literature search was performed across major electronic databases including PubMed, Scopus, and Google Scholar, as well as relevant regionally indexed Saudi journals.

Search terms included combinations of keywords such as “menopause”, “Saudi Arabia”, “primary healthcare”, “cultural beliefs”, “traditional medicine”, “psychological impact”, “telemedicine”, and “women's health”. Boolean operators (AND/OR) were used to refine search results. The search was limited to articles published in English between 1995 and 2024. This timeframe was selected to ensure inclusion of both earlier relevant literature and more recent studies reflecting evolving cultural and healthcare perspectives. The search was conducted up to the most recent complete year at the time of review.

To ensure contextual relevance, regional Saudi journals (e.g., *Journal of Family and Community Medicine* and *Saudi Medical Journal*) were also searched for peer-reviewed studies published in English addressing primary healthcare and women's health topics.

Studies were considered eligible if they addressed one or more of the following domains: menopausal symptoms, cultural or religious perceptions, traditional and complementary therapies, patient–provider communication, psychological or social dimensions, and primary healthcare system readiness within Saudi Arabia or comparable cultural contexts. Both qualitative and quantitative studies were included.

Titles and abstracts were initially screened for relevance, followed by full-text review of selected articles. The initial search yielded approximately 300 records. After screening, approximately 110 articles were reviewed in full text, of which 55 were included in the final synthesis based on relevance to the thematic domains. Additional sources were identified through manual screening of reference lists (snowballing). International clinical guidelines and position statements [e.g., NICE, ACOG, and the International Menopause Society (IMS)] were also included to contextualize clinical management approaches.

Given the narrative nature of this review, no formal quality appraisal tool or meta-analysis was performed. Instead, studies were selected based on their relevance, methodological clarity, and contribution to the overall thematic framework. Data were synthesized using a thematic approach.

A narrative review design was selected because the objective of this study was to provide a broad, context-sensitive synthesis of cultural, psychological, social, and healthcare-related dimensions of menopause within the Saudi setting rather than to answer a narrowly defined clinical question suitable for systematic review methodology. To reduce selection bias, a structured search strategy, predefined eligibility criteria addressing major menopause-related domains, and manual reference screening were used throughout the review process. Study inclusion and subsequent thematic grouping decisions were guided by relevance to the review objectives, contextual applicability to Saudi Arabia or comparable cultural settings, and methodological clarity of the included literature. Given the single-author nature of the review, duplicate independent screening was not performed; however, articles were reviewed iteratively to ensure consistency in thematic categorization and interpretation.

The thematic domains presented in this review were not predefined but emerged iteratively during the process of literature synthesis. Studies were grouped based on recurring concepts and relevance to menopause management within the Saudi context. This resulted in key themes including cultural and religious perspectives, primary healthcare readiness, patient–provider interaction dynamics, psychological and social dimensions, and culturally adapted management strategies.

This structured methodology was adopted to improve clarity, transparency, and reproducibility while maintaining the flexibility inherent to narrative reviews. It also allowed for an integrated, context-sensitive analysis appropriate for the aims of the present review (Table 1).

**Table 1:** Key thematic domains influencing menopause management in Saudi Arabia.

Major theme	Subtopic discussion
1. Cultural Context of Menopause	Religious beliefs Cultural framing of symptoms Cultural norms shape symptom expression
2. Role of Traditional & Complementary Therapies	Cultural trust in “natural” therapies is high; many women self-treat without physician oversight. Integration into care requires culturally sensitive counselling and safety monitoring.
3. Primary Healthcare System Readiness	Physician training gaps National guidelines applications
4. Patient Provider Dynamics	Communication barriers Impact on diagnosis and management
5. Psychological Dimension	Coping mechanism Stigma and associated stress
6. Social and Family Dynamics	Family role and support systems
7.Culturally Grounded Coping Mechanisms	Religious support Peer support
8. integration of culturally appropriate modalities	Community programmes Telemedicine

Key thematic areas in menopause management in Saudi Arabia.

## 3 Cultural context of menopause in Saudi Arabia

### 3.1 Cultural and religious beliefs about menopause

Traditionally, aging and its relationship with losing a women's reproductive ability has been unfavourably viewed by societies. A lot of the reported symptoms by menopausal women and their health-seeking behaviours in general during this period vary according to social background, ethnicity and religious orientation (11, 12).

For example, the most reported symptoms in the West are hot flashes. In Europe, females report sexual dysfunction more commonly while in Japan a lot of female patients report shoulder and body pains as the main bothersome symptom. Arabs in general, Saudi Arabia is no different, report hot flashes and low mood mostly. Interestingly, Sexual dysfunction and decreased libido are amongst the least reported symptoms (13).

Menopause is generally associated with relief from menstrual-related symptoms and the need for contraception; however, the way this transition is perceived and experienced varies across cultural contexts, including Saudi Arabia. In some cultural contexts, including parts of Saudi society, menopause may be perceived as a transition into a more mature social role. Some studies suggest that aging in general can be associated with increased respect and family influence; however, evidence specific to menopause-related shifts in social status remains limited and warrants further exploration (9, 10, 14).

Culturally, the increasing number of aging women may contribute to more open discussion and gradual normalization of menopause and accepting it as part of her normal evolutionary stages thus alleviating some of the mainstream negative implications that came with it such as the inability to have children and the loss of reproductive capacity (14, 15).

### 3.2 Influence of islamic teachings on menopause

Islam influences how perimenopausal and menopausal women deal with its symptoms and may also affect their decision to seek professional help (10).

A lot of Muslim women revert to prayer asking the mighty Allah for aid in dealing with pains and other bothersome symptoms. Seeking medical help is encouraged by the Islamic values and is viewed as part of the trust in Allah's well-planned goals for his worshippers. Still, less than half of menopausal women seek medical help for menopausal related symptoms primarily (10, 15). Such behaviours could be related to other factors than religious ones especially factors related to cultural norms, factors that deal with the understanding that menopause is a medical condition that requires medical attention, and other less defined factors like the ones related to healthcare systems.

### 3.3 Influence of traditional and complementary therapies

In Saudi Arabia, traditional, herbal, and complementary therapies play a prominent role in managing menopausal symptoms due to cultural, religious, and social influences. Many Saudi women prefer natural and religious approaches over conventional hormone replacement therapy (HRT). These traditional remedies are viewed as considerably safe and culturally appropriate (16, 17).

Commonly used herbal remedies include fenugreek (Helba), flaxseed, black cohosh, sage, and anise. These herbs are a common staple in the Saudi cultural contexts and are believed to help with hot flashes, night sweats, and mood changes. Other popular procedural methods include cupping therapy with or without the superficial cutting of the epidermal layer or what is called (hijama), spiritual practices such as ruqyah (Qur'anic healing) and traditional deep massages from traditional healers. Increasing soy-rich foods has also gained popularity as well but not as commonly seen as in the far eastern societies (16, 17).

Research in Saudi Arabia supports the widespread use of these methods. A 2015 study conducted in Riyadh found that nearly 60%–70% of menopausal women reported using herbal or traditional remedies to manage their symptoms, while only about 15%–20% used HRT. Similarly, a 2020 study in Jeddah reported that over 65% of women preferred herbal treatments, often obtained from family, friends, or local herbal shops rather than healthcare providers. The main reasons cited were fear of side effects of HRT, cultural acceptability, and perceived natural safety (18, 19).

Despite their popularity, researchers caution that many women use these therapies without medical supervision, and scientific evidence regarding their safety and efficacy remains limited. Health professionals in Saudi Arabia are therefore encouraged to integrate culturally sensitive health education, addressing misconceptions while respecting traditional beliefs, to help women make informed choices about menopausal management.

## 4 Primary healthcare and menopausal management

### 4.1 Readiness of PHC services

Studies consistently show that many PHC physicians in Saudi Arabia have limited training and awareness regarding menopause management. A study in Riyadh Al-Ghamdi et al. (14) found that only about one-third of PHC physicians had received any formal education on menopause or hormone replacement therapy (HRT) (14). Another study in Jeddah revealed knowledge gaps in diagnosing and counselling menopausal women, with most providers relying on personal experience rather than national or international guidelines (15).

PHC centers rarely have specialized professionals, and care is often handled by general practitioners and family physicians. Continuing medical education (CME) programs focusing on women's midlife health are limited and not standardized across PHCs as well.

### 4.2 Medical approach in PHC: gaps and opportunities

According to the Saudi Ministry of Health (MOH) primary care model (2021), PHC centres prioritize maternal and reproductive health, chronic disease, and preventive services, but specific menopause clinics or referral pathways are scarce although possible (20). Currently, Saudi Arabia does not have a national menopause management guideline integrated into PHC practice. Most clinicians rely on international protocols (e.g., NICE, ACOG guidelines).

The Saudi Society of Obstetrics and Gynaecology (SSOG) has discussed the need for a national framework for midlife women's health, but implementation remains limited (21). In contrast, national initiatives such as Vision 2030 and the Saudi Health Sector Transformation Program emphasize women's health empowerment, which provides an opportunity to integrate menopause care into preventive and wellness programs (22).

Few structured menopause education or counselling programs exist at PHC level. Studies in different Saudi regions show that most PHC centres lack educational materials or awareness campaigns targeting midlife women. However, some hospitals and teaching institutions around the Kingdom, that have a little more autonomy with the new hierarchical distribution of health clusters in Saudi Arabia have begun small-scale women's midlife health clinics and CME workshops to improve primary care readiness (23–25).

## 5 Patient–provider interaction dynamics and their influence on menopausal management

### 5.1 Gender dynamics in healthcare encounters

Cultural norms in Saudi Arabia strongly influence interactions between women and healthcare providers. Many Saudi women prefer female physicians, especially for reproductive or intimate health issues such as menopause (26). Studies show that women feel embarrassed or reluctant to discuss menopausal symptoms—particularly sexual, psychological, or urogenital issues—with male doctors (14, 15). In rural or conservative regions, the shortage of female physicians in PHC centres often leads to underreporting or complete avoidance of menopause-related consultations (27). Some women instead seek advice from family members, friends, or traditional healers, further limiting the role of medical professionals.

### 5.2 Barriers to effective communication

Several systemic and cultural barriers limit open and effective communication between women and PHC providers (28). These are especially prominent when discussing topics related to menstruation, sexual health, and mood which are often considered private or “shameful” to discuss openly, especially with male or younger providers. These barriers may be further influenced by cultural norms in Saudi Arabia and the general modesty of women, nevertheless, it is an observed pattern worldwide as well (28, 29).

Lack of time and privacy in busy PHC clinics often lead to brief consultations focused on physical symptoms rather than holistic management (30).

Knowledge and attitude gaps among providers indicate that many PHC doctors feel uncomfortable initiating menopause discussions due to limited training and uncertainty about treatment options, which further discourages patient openness (30–32).

Many Saudi women, like other nations, interpret menopausal symptoms as a natural stage rather than a health concern requiring medical attention, this is mostly due to the low level of awareness resulting in low health-seeking behaviour (10, 33).

### 5.3 Influence on menopause management

The factors contributing to deficiencies in menopause management are often multifactorial and reflect the interaction of the previously described dynamics.

These identified factors, as we have earlier specified, could be related to health systems, patients' beliefs, cultural norms as well as health provider factors. Collectively, they lead to delayed diagnosis and limited use of evidence-based treatments such as HRT or behavioural interventions. This may contribute to increased reliance on herbal and traditional remedies due to the public's trust in informal networks over clinical advice (11, 12).

## 6 Psychological and social dimensions of menopause in Saudi women

The literature shows that menopause in Saudi women is not only a biological transition but also a social and emotional experience shaped by family roles, gender expectations, and coping traditions.

### 6.1 Psychological well-being

Menopause is often associated with psychological symptoms such as mood swings, irritability, anxiety, sleep disturbances, and depression (34, 35). In Saudi Arabia, studies indicate that the psychological impact can be significant, particularly when menopause is accompanied by limited awareness or social support. Studies emerging from big cities in Saudi Arabia report staggering numbers that could go up to as high as 60% when it comes to mood changes, and about 30%–40% reported anxiety or depressive symptoms (34). The emotional distress that accompanies menopause is often augmented by the lack of open discussion on the condition and the perceived end of fertility and loss of femininity that comes with it (36).

Women with higher education show better coping and lower psychological distress. This aligns with international data indicating improved outcomes perceived personally or by healthcare providers in women with higher educational status and self-esteem at the beginning of menopause (35, 36).

### 6.2 Coping mechanisms

Saudi women employ a range of culturally grounded coping strategies. Religious and spiritual coping are most common. Practices such as prayer, Qur'an recitation, and acceptance of menopause as “Allah's will” provide psychological comfort and resilience (18, 37).

Social support from family, especially female relatives and friends, plays a critical role in emotional adjustment. We have stated before that many women in Saudi Arabia normalize menopause as a natural stage, focusing on its positive aspects like freedom from menstruation or pregnancy risk making coping with it much easier (35, 38). Turning into herbal remedies as explained earlier is another established coping mechanism in Saudi women allowing them to self-care in a traditional way (16).

Primary health care services theoretically focus on mental health of patients. Mental health importance is intensely reflected in PHC from MOH Vision 2030 reform initiatives; however, whether these are effectively implemented truly applied is another issue. Lack of access to mental health support or trained counsellors in PHC settings limits women's ability to receive professional psychological guidance. This focuses on a huge opportunity for future menopausal health initiatives (39).

### 6.3 Social and family dynamics

In the Saudi context, where family and marital roles are highly valued, menopause can influence spousal relationships and family dynamics. Some women report reduced intimacy or communication with their spouses due to symptoms such as vaginal dryness, low libido, or mood swings. These topics are often considered too private to discuss openly (10, 17).

A study in the Eastern Province of Saudi Arabia noted that about half of menopausal patients participating in the study experienced a perceived decline in marital satisfaction, though many attributed it to aging rather than menopause itself (26).

Conversely, some women reported perceptions of increased respect or family influence as they transitioned into a “mature” or “wise” role, which can improve emotional well-being (17, 40).

Keeping in mind that spousal and family support is a protective factor against declining mental health in menopausal women, women who receive understanding and empathy from their husbands and family members show better adjustment and fewer depressive symptoms (40).

### 6.4 Cultural framing of menopause

Saudi society often views menopause through a moral and spiritual lens, emphasizing acceptance and patience. Menopause is generally not pathologized but seen as a normal life stage, though silence and modesty around reproductive topics mean many women endure symptoms privately. The lack of open dialogue in families and healthcare settings can reinforce feelings of isolation (41).

Urban, educated women, who represents a growing demographic in the Saudi society, are increasingly challenging these taboos, seeking medical and psychological support and engaging in health education initiatives. These small but steady movements happening in urban big cities prominently so more than in smaller remote areas where menopausal ladies feel more isolated by the healthcare system and find refuge in social gatherings engaging in experience exchange with other socially compatible menopausal women (42, 43).

## 7 Integration for culturally appropriate menopause management

Culturally based modalities such as herbal remedies, community programs, and telemedicine may contribute to improving access, acceptability, and patient engagement in menopausal care among Saudi women. When integrated within evidence-informed and culturally sensitive care models, these approaches may help address stigma, encourage patient participation, and support holistic well-being aligned with Saudi cultural and religious values (44).

In Saudi Arabia, health behaviours and treatment choices are strongly influenced by cultural beliefs, religious values, and social norms. Integrating culturally appropriate modalities into menopause management may increase trust, communication, and patient engagement with healthcare services. Approaches that acknowledge traditional views on femininity, modesty, and aging may encourage more open discussion of menopausal concerns and greater willingness to seek professional support (10).

### 7.1 Use of traditional and herbal remedies

As previously discussed, many Saudi women rely on traditional herbal and dietary remedies for managing bothersome menopausal symptoms. These remedies represent accessible, familiar, and culturally accepted approaches, particularly for women hesitant about hormone replacement therapy (HRT) (16). Although some herbal therapies have shown potential benefits in symptom management, available evidence regarding their effectiveness and safety remains variable. Accordingly, integration of such practices into clinical care should be approached cautiously through evidence-based validation, safety monitoring, and physician awareness to minimize possible interactions with prescribed medications. Encouraging open discussions between healthcare providers and patients regarding herbal use may help bridge the gap between traditional practices and modern medicine while promoting informed decision-making and patient trust (45–47).

### 7.2 Community-based support programs

Culturally sensitive community-based initiatives, such as women's health education groups, mosque-based wellness sessions, or local primary healthcare centre programs, have been proposed as supportive approaches for menopause awareness and peer engagement (48). These initiatives may provide women with opportunities to share experiences and discuss coping strategies within culturally comfortable settings. Some literature suggests that community-based approaches may help reduce stigma, improve awareness, and encourage psychosocial support surrounding menopause-related concerns (48). Programs led by female healthcare professionals or community health workers may also be more culturally acceptable within certain Saudi communities and may facilitate discussions that incorporate religious and social dimensions of health (49, 50).

### 7.3 Role of telemedicine and digital health platforms

The inclusion of telemedicine within this review reflects its expanding role in healthcare delivery in Saudi Arabia, particularly within Vision 2030 health transformation initiatives. Telemedicine may offer a potentially useful approach to addressing barriers related to privacy, gender preferences, and geographical access in conservative or underserved settings, making it a culturally compatible extension of contemporary healthcare models (51). Telemedicine services in Saudi Arabia have expanded considerably under the national eHealth strategy. Within menopause care, digital consultations, remote follow-up services, and online educational resources may provide additional avenues for healthcare access, particularly for women who face barriers to in-person consultations. Arabic-language digital health resources may also support symptom awareness, health education, and patient engagement (52).

An additional potential advantage of telemedicine platforms is the facilitation of communication between female patients and female healthcare providers through secure remote consultations. This approach may help reduce gender-related communication barriers and improve consultation comfort for some women (53, 54).

## 8 Summary

Menopause among Saudi women is a multidimensional experience involving biological, psychological, and social changes. Psychological distress is common but often underrecognized due to cultural norms that discourage emotional expression or help-seeking.

Coping strategies are largely faith-based and socially rooted, highlighting the importance of religious, familial, and community support. Addressing menopause holistically including its emotional and relational aspects is essential for improving women's well-being and quality of life in Saudi Arabia.

### 8.1 Recommendations and call for action

Overall, Saudi PHC services are in an early stage of readiness for managing menopause. There is growing awareness but limited training, absence of national guidelines, minimal specialized services, and few structured programs. The following are improvement strategies and areas of growth that can truly impact menopausal management within the Saudi context:

- Strengthening CME, developing national clinical protocols, and integrating menopause management into women's health initiatives are key steps to improving readiness.
- Saudi and international literature propose several culturally appropriate strategies to improve patient–provider dynamics such as increasing the number of trained female PHC providers in women's health and menopause management.
- Cultural competence training for all healthcare workers to help them approach menopause sensitively and respectfully.
- Developing private counselling spaces in PHC centres to encourage open discussions.
- Incorporating communication skills and gender sensitivity into CME for PHC providers.
- Community-based education programs that normalize menopause as a health topic, empowering women to seek professional help.
- Encouraging shared decision-making approaches, where women are active participants in treatment choices, may improve satisfaction and engagement with care.

Integrating these modalities may support a biopsychosocial model of menopause management that honours cultural identity while maintaining scientific rigor. It enhances patient-centred care, improves adherence, and bridges traditional wisdom with modern healthcare. Collaboration between healthcare professionals, traditional healers, and community leaders can ensure that interventions are safe, ethical, and effective.
